# Tooth-size discrepancy: A comparison between manual and digital methods

**DOI:** 10.1590/2176-9451.19.4.107-113.oar

**Published:** 2014

**Authors:** Gabriele Dória Cabral Correia, Fernando Antonio Lima Habib, Carlos Jorge Vogel

**Affiliations:** 1 Specialist in Orthodontics and Facial Orthopedics, Federal University of Bahia (UFBA).; 2 Assistant professor, UFBA.; 3 PhD in Orthodontics, University of São Paulo (USP).

**Keywords:** Dental models, Computer-assisted diagnosis, Three-dimensional imaging

## Abstract

**Introduction:**

Technological advances in Dentistry have emerged primarily in the area of diagnostic tools. One example is the 3D scanner, which can transform plaster models into three-dimensional digital models.

**Objective:**

This study aimed to assess the reliability of tooth size-arch length discrepancy analysis measurements performed on three-dimensional digital models, and compare these measurements with those obtained from plaster models.

**Material and Methods:**

To this end, plaster models of lower dental arches and their corresponding three-dimensional digital models acquired with a 3Shape R700T scanner were used. All of them had lower permanent dentition. Four different tooth size-arch length discrepancy calculations were performed on each model, two of which by manual methods using calipers and brass wire, and two by digital methods using linear measurements and parabolas.

**Results:**

Data were statistically assessed using Friedman test and no statistically significant differences were found between the two methods (P > 0.05), except for values found by the linear digital method which revealed a slight, non-significant statistical difference.

**Conclusions:**

Based on the results, it is reasonable to assert that any of these resources used by orthodontists to clinically assess tooth size-arch length discrepancy can be considered reliable.

## Abstract (pt)

**Introdução:**

na Odontologia, os avanços tecnológicos vêm se manifestando, principalmente, em instrumentos de diagnóstico, como o desenvolvimento dos *scanners* 3D, capazes de transformar modelos de gesso em modelos digitais tridimensionais.

**Objetivo:**

o objetivo da presente pesquisa foi avaliar a confiabilidade da análise da Discrepância de Modelo realizada em modelos digitais tridimensionais, comparando-a com a obtida em modelos de gesso.

**Métodos:**

utilizou-se modelos de gesso das arcadas dentárias inferiores e seus correspondentes modelos digitais tridimensionais, adquiridos por meio do *scanner* 3Shape R700T. Foram realizados quatro diferentes cálculos da Discrepância de Modelo para cada modelo selecionado, dois desses por meio de métodos manuais, utilizando paquímetro e fio de latão, e dois por meio de métodos digitais, utilizando medições lineares e por meio da confecção de uma parábola.

**Resultados:**

os dados obtidos foram avaliados estatisticamente por meio do teste de Friedman, e observou-se não haver diferença estatisticamente significativa entre os métodos utilizados (p > 0,05), exceto os valores obtidos pelo método digital linear, onde observou-se uma pequena diferença estatística, porém, não são valores considerados clinicamente significativos.

**Conclusão:**

com base nos resultados, é possível afirmar que, quaisquer desses recursos que o ortodontista venha a utilizar em sua vida clínica para obtenção da Discrepância de Modelo, esses são considerados métodos confiáveis.

## INTRODUCTION

Plaster models provide a three-dimensional view of occlusion, allowing professionals to assess in greater detail the impressions obtained during clinical examination without interference from soft tissues of the mouth, which facilitates the study of a case.^1,2,3^ Thus, the use of models is vital if one is to reach an accurate diagnosis.^1,4-9^

With the aid of orthodontic models, the clinician can assess symmetry, arch form, severity of the curves of Spee and Wilson, axial inclinations as well as perform analyses such as Peck and Peck, Bolton and Tooth Size-Arch Length Discrepancy.^10^

Tooth size-arch length discrepancy (TSALD), or Space Analysis, plays a pivotal role in orthodontic practice, being represented by the following formula: TSALD = Space Available - Space Required, where the Space Available (SA) represents the basal region available in the dental arch, and the Space Required (SR) is equal to the sum of mesiodistal diameters of existing teeth. Diagnostic decisions to determine whether extractions are necessary to accommodate teeth in the dental arch are usually made based on this evaluation along with other factors such as cephalometric and profile analyses.^1,2,5,11,12^

Lately, there have been significant advances in computer science of which effects have been increasingly felt in different areas of dental practice. In Orthodontics, these advances have been particularly significant in terms of diagnostic tools.^3,13,14^

Similarly to digital photographs and radiographs developed to replace their analogical counterparts, thereby facilitating diagnosis and interdisciplinary planning, plaster model scanning has also gained momentum through the development of 3D scanners.^1,4,6,15^ According to Dubón,^11^ these resources have been widely employed in orthodontic practice.

Three-dimensional scanners are devices used to convert volumetric objects into three-dimensional digital images. In other words, they analyze a real-world object and collect data on its shape and appearance, turning it into a three-dimensional digital file.^16^ Three-dimensional plaster model scanning is advocated by many authors as a viable option to overcome existing limitations in handling traditional plaster casts. Furthermore, they allow clinicians to obtain measurements of models more rapidly.^5,17-22^

Different technologies have been developed to build 3D scanning devices, each with their own limitations, advantages and disadvantages.^1^ Thus, in view of ongoing technological advances, the many benefits offered by recent resources and the availability of several different brands of 3D scanners in the market - such as 3Shape's R700^TM^ scanner and its associated software OrthoAnalyzer^TM^ - it becomes necessary to assess the reliability of tooth size-arch length discrepancy measurements performed on three-dimensional digital models and compare these measurements with those obtained from plaster models by means of traditional methods.

## MATERIAL AND METHODS

This study was approved by the Federal University of Bahia (UFBA) Institutional Review Board under protocol #235,134, and registered by the Brazilian National Research Ethics Committee (CONEP) under CAAE 12489513.2.0000.5024.

This study adopts an experimental approach using plaster models (casts) and three-dimensional digital models captured with a R700TM scanner manufactured by 3shape, and measurements performed with 3Shape OrthoAnalyzer^TM^ software, a digital caliper and brass wire.

### Characterization of the sample

Thirty mandibular casts of patients in the initial phase and with permanent dentition were selected. Inclusion criteria were as follows:

- Absence of positive or negative bubbles.

- Presence of all teeth, from #36 to 46.

- Teeth in perfect condition, with no anatomical defects.

### Tooth size-arch length discrepancy

Four calculations were performed for each patient.

Two calculations by manual methods:

» **TSALD 1 = SA (caliper) - SR**

» **TSALD 2 = SA (brass wire) - SR**

And two calculations by digital methods:

» **TSALD 3 = SA (linear) - SR**

» **TSALD 4 = SA (parabola) - SR**

### Model manipulation

#### Obtaining manual measurements

Material used to obtain manual measurements not only included plaster models, but also index cards for data recording, a Cen-Tech 4-in digital caliper (Harbor Freight Tools, Calabasas, CA, USA) with 0.01 mm accuracy, brass wire, a ruler, pencil and eraser.

**Figure 1:**
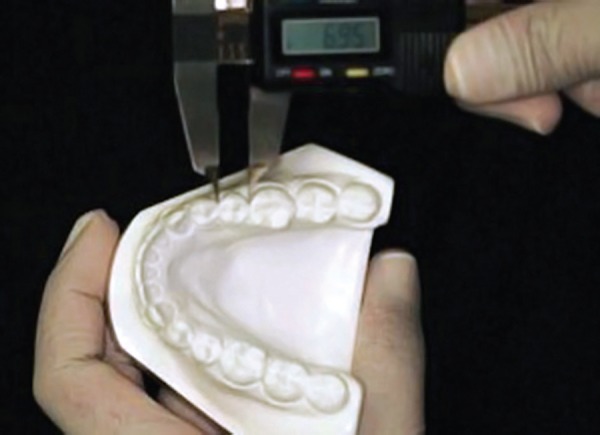
Space Required - Measuring the mesiodistal width of second right premolar.

Initially, the space required (SR) was recorded with the aid of a digital caliper. Each value was obtained by placing the caliper over the largest mesiodistal width of lower teeth, starting from the second right premolar (Fig 1), followed by the first premolar, canine and incisors on the same side. The procedure was repeated on the left side, starting from the incisor, followed by the canine, first and second premolars. Measurements were recorded on the index cards.

**Figure 2:**
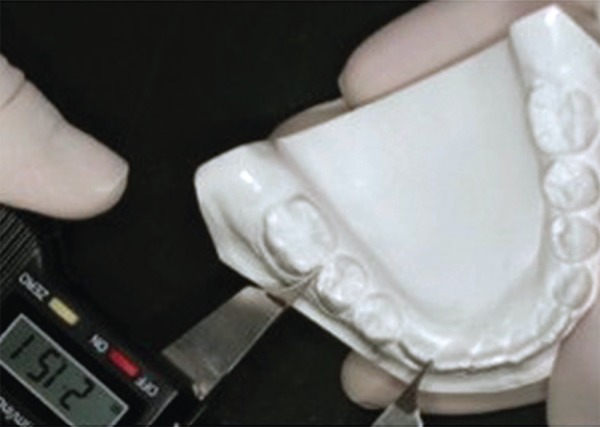
Space Available - Caliper positioned from the mesial surface of first right molar to the mesial surface of right canine.

**Figure 3:**
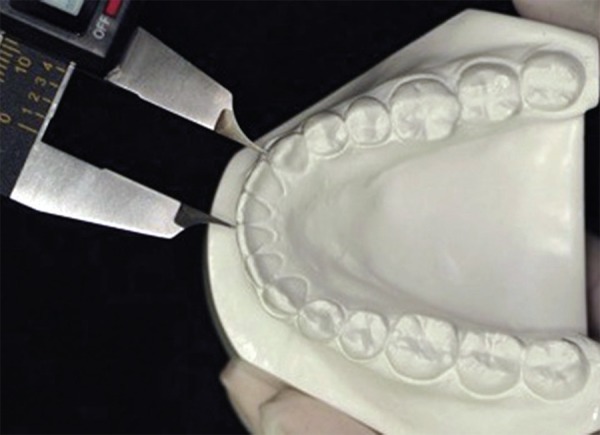
Space Available - Caliper positioned from the mesial surface of right canine to the region between central incisors.

After this step, the space available (SA) was measured, which corresponds to the size of the basal bone lying between the mesial surface of the first permanent molar on one side to the mesial surface of the first permanent molar on the opposite side. This measurement was obtained by two different methods. The first involved the use of a digital caliper to measure the SA. Thus, the caliper was positioned from the mesial region of the lower right first molar to the mesial surface of the canine (Fig 2), and from the mesial surface of the canine to the region between central incisors (Fig 3). The same procedure was carried out on the opposite side, starting from the region between incisors.

**Figure 4:**
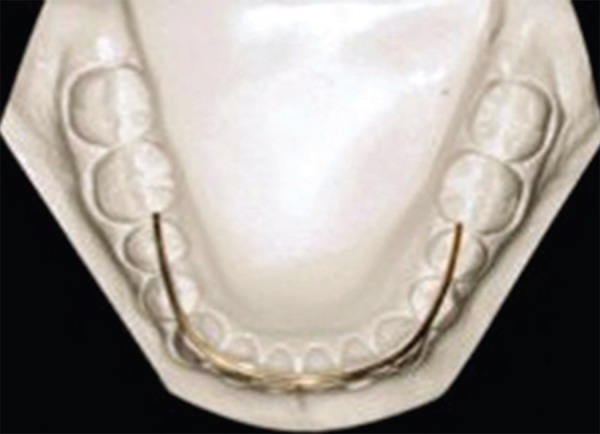
Measuring Space Available with brass wire.

After being recorded with a digital caliper, SA was measured with the aid of brass wire. A parabola was, therefore, formed with brass wire contouring the central grooves of the occlusal surfaces of posterior teeth and the incisal surfaces of anterior teeth, from the mesial surface of the mandibular first molar to the mesial surface of the lower left first molar (Fig 4). The brass wire was then straightened out, and with the aid of a steel millimeter ruler, the value for SA was ultimately obtained.

Finally, space analysis calculations were performed, the first using SA measurements obtained with the digital caliper [TSALD = SA (caliper) - SR], and the second using the values obtained with the brass wire [TSALD = SA ( brass wire) - SR].

All measurements were performed by a single observer (T_1_) previously trained. 20% of the models were measured again after 15 days (T_2_) so as to test the operator's calibration.

#### Obtaining the digital model

Model scanning was performed with a 3Shape R700TM scanner (Copenhagen, Denmark). The device consists of a platform with support for the models, a laser and two digital cameras that capture high resolution images.

Scanning is performed with this equipment using a non-destructive laser beam which reproduces model surfaces so that the plaster model is not discarded.

First, the lower model was positioned on the platform so that the 3D scanner laser beam could map the desired profile. To this end, the platform is automatically rotated and inclined during scanning, thereby ensuring complete coverage of the object's geometry.

For the scanning process, it is necessary to start the ScanItOrthoImpression computer program and register the patients. Scanning is started after recording the patients' data and positioning the model on its base. During this process, the points on the plaster model are captured by the laser, thus rendering the virtual image. Images are obtained by organizing the points in a triangular form. The virtual image file is saved in DICOM format.

Once the model images are captured, 3Shape OrthoAnalyzer^TM^ software (Copenhagen, Denmark) runs for manipulation of the digital model. Prior to model scanning, the scanner is calibrated with the aid of two tables which are attached to the scanner in order to standardize the measurements. This care was taken daily until the full digital sample was obtained.

#### Obtaining digital measurements

**Figure 5:**
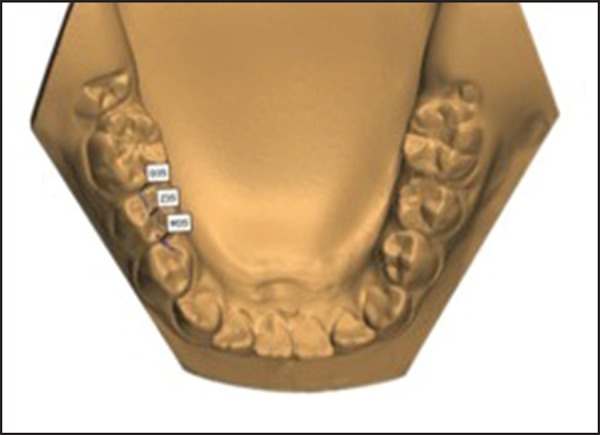
Space Required - Measuring the mesiodistal width of second right premolar on the virtual model.

The digital models were measured with OrthoAnalyzer^TM^ software. Initially, landmarks were plotted on the widest possible mesiodistal distance between teeth, starting from the second lower right premolar to the second lower left premolar, tooth after tooth, in order to record the SR values (Fig 5). Similarly to the manual method, SA measurements were performed using two different techniques: One using linear values, and one by forming a parabola.

**Figure 6:**
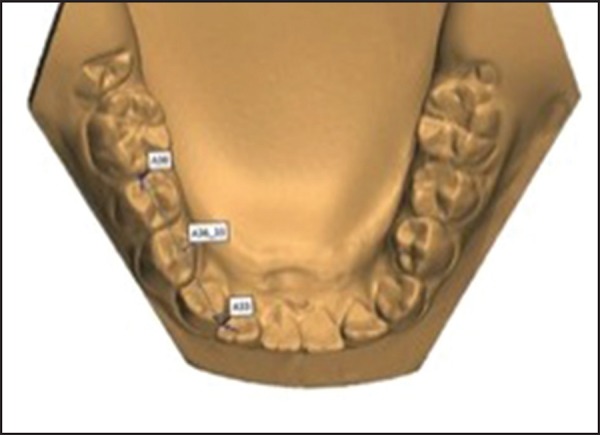
Space Available - Landmarks plotted from the mesial surface of first right molar to the mesial surface of right canine on the virtual model.

**Figure 7:**
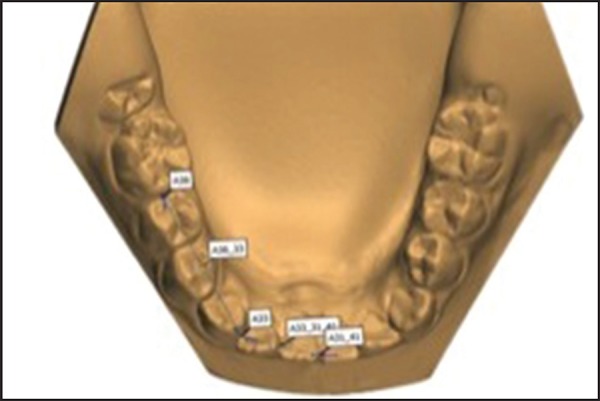
Space Available - Landmarks plotted from the mesial surface of right canine to the region of central incisors on the virtual model

Thus, linear values were used first in order to perform SA measurements. The procedure involved plotting the landmarks from the mesial surface of the mandibular right first molar to the mesial surface of lower right canine (Fig 6), and from the mesial surface of the canine to the region between central incisors (Fig 7). The same procedure was adopted for the opposite side, with plotting starting from the region between incisors.

**Figure 8:**
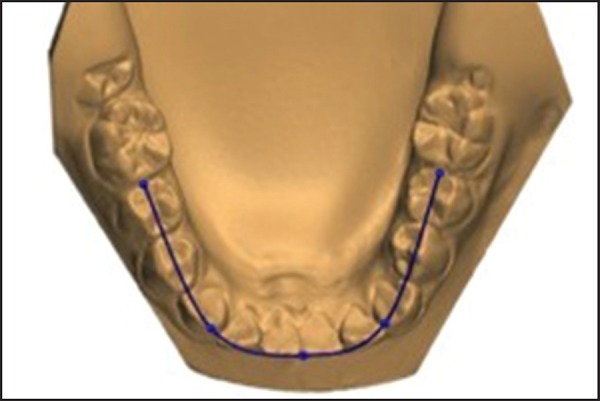
Measuring Space Available on the virtual model with the aid of a parabola.

In order to obtain SA values by means of a parabola, the parabola had to be formed in such a way as to contour the occlusal surfaces of posterior teeth and incisal surfaces of anterior teeth (Fig 8).

Finally, two tooth size-arch length discrepancy calculations were performed. The first, using SA measurements obtained through linear measurements [TSALD = SA (linear) - SR], and the second using the values obtained when shaping the parabola [TSALD = SA (parabola) - SR].

All digital measurements as well as those obtained by the manual method were performed by a single observer previously trained (T_1_). 20% of the models were measured again after 15 days (T_2_) so as to test the operator's calibration.

## STATISTICAL ANALYSIS

Intraclass correlation coefficient (ICC) statistic was performed using BioEstat 5.0 software to assess the operator's calibration. Once found, TSALD values were compiled into a spreadsheet and subjected to statistical analysis, whereas Kolmogorov-Smirnov test was applied to assess data normality. Furthermore, reliability between methods was tested by Friedman test using BioEstat version 5.0 software (Bélem, PA - Brazil).

## RESULTS

Excellent reproducibility (0.98) of methods was found after intraclass correlation coefficient (ICC) statistic, which is considered indicative of optimal agreement between measurements obtained at T_1_ and T_2_ with the operator duly calibrated for research.

Komogorov Smirnov statistical test confirmed the hypothesis of an abnormal distribution between T_1_ and T_2_ if the median and quartiles (25% and 75%) were employed as measures of variability (Table 1).

**Table 1:** Values of medians and 25% and 75% interquartile interval values.

Method		25%	Median	75%
Manual	TSALD caliper	-1.48	-0.41	1.89
	TSALD brass wire	-1.40	-0.60	2.44
Digital	TSALD linear	-0.88	-0.15	2.52
	TSALD parabola	-1.02	-0.38	2.07

**Figure 9:**
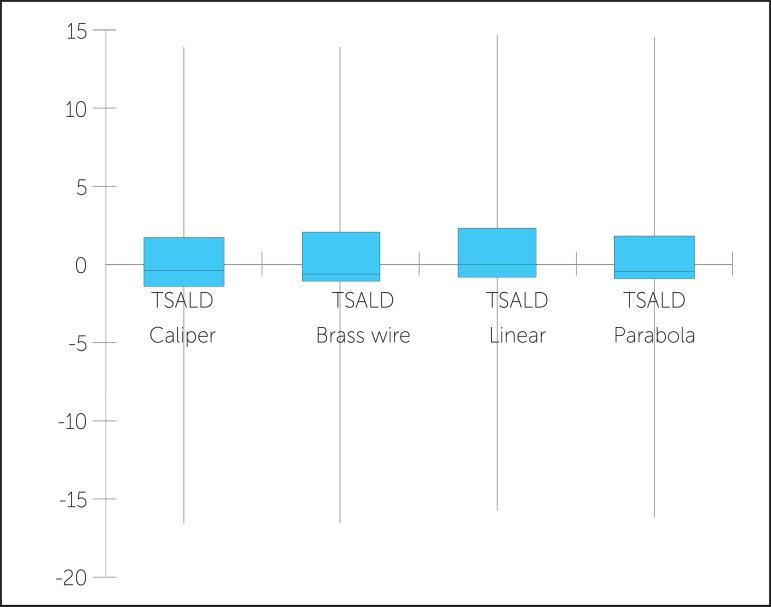
Box plot with median values of the four different methods used to obtain tooth size-arch length discrepancy.

Friedman statistical test was performed to assess method reliability, which showed (at a significance level of P ≤ 0.05) that the differences between measurements were not statistically significant, except for the values found by the linear digital method (Fig 9) which revealed a slight, non-significant statistical difference. This finding also allows one to assert that these methods, used by orthodontists in their professional life to obtain tooth-size discrepancy,can be considered reliable.

## DISCUSSION

Given the remarkable development of computer science, which arises increased interest in 3D images among orthodontists, a number of companies currently offers services to transform plaster casts into three-dimensional digital models.^1,3,8,14,20^

This method has several advantages, namely: reduced physical space, averting the risk of breakage, easy data storage, simultaneous exchange of information with colleagues, and greater efficiency and productivity in dental practice.^6,15-22^

However, despite all these advantages, the exclusive use of digital models in daily practice is not yet routine as it also features some disadvantages in its application, namely: data loss in case of degradation of electronic storage, dependence on third parties, time-consuming software support, need to learn the operating system, and high cost of equipment.^6,21^

Faced with these new technologies, some authors have assessed the reliability of these new methods. Alcan et al^23^ and Sousa et al^24^ conducted a research to assess the reliability of measurements performed on digital models obtained with a D250 scanner, manufactured by 3Shape. The authors found no statistically significant difference between measurements obtained directly on the plaster models *versus* the digital models. They concluded, therefore, that digital models can be used in orthodontic practice thanks to their accuracy and reproducibility.

Leifert et al^25^ assessed SA and SR measurements on dental casts using a digital caliper and brass wire, and on three-dimensional digital models using OrthoCad software. They observed that scanning accuracy required for spatial analysis on digital models is clinically acceptable and reproducible when compared with traditional plaster model analysis. These results agree with those of the present study, since there was no difference between manual and digital methods, although OrthoAnalyzer^TM^ software was employed to obtain digital measurements.

Grehs^26^ assessed the accuracy and reproducibility of tooth size and interdental measurements taken on plaster *versus* digital models scanned with a 3Shape R-700TM Scanner. A digital caliper was used for the manual measurements whereas O3d (Widialabs) software was used for the digital measurements. Results revealed that both the caliper and the O3d software had similar performance in enabling measurements and analyses. Although research assessed different measurements, the results agree with those of the present study, as it became clear from the methodology developed herein that there is similarity between measurements obtained manually and digitally.

Further corroborating these results, the investigation conducted by Zilberman et al,^5^ Quimby et al^1^ and Bootvong et al^27^ compared plaster and digital (OrthoCad) models, and showed that both methods are effective and can be reproduced when measuring tooth size and dental arch widths. Quimby et al^1^ also suggested that features such as convenient storage and shorter time required for measuring with the digital system are likely to render this method attractive for orthodontists.

However, Garino and Garino^17^ found statistically significant differences between measurements obtained with digital *versus* plaster models using OrthoCad software, thereby disagreeing with the results of this study which found that both digital and manual measurements are just as reliable. Furthermore, it is noteworthy that the authors cited above used an analog caliper with 0.5 mm precision to obtain the manual measurements and an instrument with 0.1 mm precision to perform the digital measurements, which discloses the limitations of measuring a manual analog method. In addition, tooth position such as inclination, rotation and crowding, may have influenced the measurements, thus showing significant differences, especially in plotting mesiodistal landmarks.

Research by Santoro et al^6^ also found statistically significant differences in comparing overbite and tooth size values using manual *versus* digital (OrthoCAD) methods. The authors found that digital measurements were smaller than manual measurements, and explained that the differences were probably due to an intrinsic difference between methods, such as the ability to enlarge the image to better observe the mesiodistal diameters provided by the digital method. The values, however, were not considered clinically significant.

## CONCLUSIONS

Based on the results it is reasonable to conclude that Tooth Size-Arch Length Discrepancy values found by manual (Cen-Tech digital caliper and 4-in brass wire) *versus* digital (3Shape R-700TM / 3Shape OrthoAnalyzer^TM^) methods did not differ, except for the values found by the linear digital method. The latter revealed a slight, non-significant statistical difference, thereby confirming that any of these resources used by orthodontists to clinically obtain Tooth Size-Arch Length Discrepancy can be considered as reliable methods.
